# Multi-parental fungal mapping population study to detect genomic regions associated with *Pyrenophora teres* f. *teres* virulence

**DOI:** 10.1038/s41598-023-36963-y

**Published:** 2023-06-16

**Authors:** Buddhika A. Dahanayaka, Anke Martin

**Affiliations:** Centre for Crop Health, University of Southern Queensland, Toowoomba, QLD 4350 Australia

**Keywords:** Quantitative trait, Fungal genetics

## Abstract

In recent years multi-parental mapping populations (MPPs) have been widely adopted in many crops to detect quantitative trait loci (QTLs) as this method can compensate for the limitations of QTL analyses using bi-parental mapping populations. Here we report the first multi-parental nested association mapping (MP-NAM) population study used to detect genomic regions associated with host-pathogenic interactions. MP-NAM QTL analyses were conducted on 399 *Pyrenophora teres* f. *teres* individuals using biallelic, cross-specific and parental QTL effect models. A bi-parental QTL mapping study was also conducted to compare the power of QTL detection between bi-parental and MP-NAM populations. Using MP-NAM with 399 individuals detected a maximum of eight QTLs with a single QTL effect model whilst only a maximum of five QTLs were detected with an individual bi-parental mapping population of 100 individuals. When reducing the number of isolates in the MP-NAM to 200 individuals the number of QTLs detected remained the same for the MP-NAM population. This study confirms that MPPs such as MP-NAM populations can be successfully used in detecting QTLs in haploid fungal pathogens and that the power of QTL detection with MPPs is greater than with bi-parental mapping populations.

## Introduction

To understand the evolution of plant fungal pathogenicity, it is crucial to identify the genomic regions underlying the pathogenicity^1^. Initially, it was suggested that the pathogenicity of plant fungal pathogens was due to a single or a few genomic regions/genes and that these genes were recognised by the host plant and hence, follow a gene-for-gene model^2^. With the identification of several other host–pathogen interaction model systems, the expression of virulence of plant fungal pathogens on host plants was proposed to be a cumulative effect of multiple interacting genomic loci or quantitative trait loci (QTLs)^3,4^.

The QTLs responsible for inducing a virulence response in the host plant can be dissected through QTL mapping to extract important information on the genetic architecture of these phenotypic expressions^5,6^. QTL mapping studies are performed by linkage analysis in segregating bi-parental mapping populations^5^. The number of recombination events that occur within the mapping population determines the density of the linkage map. Many bi-parental mapping population studies have been conducted in fungal plant pathogens to identify QTLs responsible for the virulence of the pathogen^7–10^. However, in bi-parental mapping populations, the number of recombination events is restricted due to inadequate genetic diversity present between the two parents resulting in detecting broad chromosomal regions responsible for the phenotypic expression^11–13^. Hence, QTL mapping in bi-parental mapping populations is limited by low genetic map resolutions, inadequate genetic diversity between parents^14^ and inability to identify the same QTL in other genetic backgrounds^15^.

To overcome the constraints of QTL mapping in bi-parental populations, genome‐wide association mapping studies (GWAS) have been used to identify QTLs responsible for the virulence of fungal plant pathogens using genetically diverse populations from unknown kinship backgrounds^16^. The high mapping resolution in GWAS is achieved by its capacity to explore a broad genetic background coupled with capturing more allelic diversity occurring through historical recombination events and employing diversity panels which may comprise of fungal isolates collected from different geographical areas, years and hosts^11,16,17^. GWAS does, however, have its own constraints, such as spurious associations from the confounding effects of inherent population stratification and cryptic relatedness. While regulating these issues is possible with certain statistical methods, the methods have less power for QTL detection associated with population structure due to false negatives.

Multi-parental mapping (MPP) methods integrate bi-parental QTL and GWAS to detect robust genomic regions associated with phenotypic traits^18^. A population for a commonly used MPP method like nested association mapping (NAM) is developed by crossing a common parent/parents with a diverse set of founder lines^15,18^. In multi-reference (MR) NAM population studies, multiple parental lines are inter-crossed without using a common parent to increase the allelic diversity and increase rare alleles to moderate frequencies^18^. Multi-parent advanced generation inter-cross (MAGIC) is another widely used MPP in which several founder lines are intercrossed over several generations^19^. Multi-connected bi-parental mapping populations used in MPP analyses increase the allelic diversity of the populations and capture both recent and historical recombination events whilst reducing the confounding effects occurring due to the population structure of the population^20^. As per the authors’ knowledge MPP studies on plant fungal pathogens have not been attempted.

Net blotch is an economical important foliar disease of barley (*Hordeum vulgare* L.)^21^ caused by the plant fungal pathogen *Pyrenophora teres* [syn: Drechslera teres]^22^. *Pyrenophora teres* is a haploid heterothallic ascomycetous fungus. Sexual reproduction in *P. teres* is controlled by a single mating type locus (*MAT1*), which exists as two alternative forms or idiomorphs, i.e. *MAT1-1* and *MAT1-2*. For the successful sexual reproduction of the fungus, two opposite mating types are required^23^. *Pyrenophora teres* exists as two forms, *P. teres f. teres* (*Ptt*) and *P. teres f. maculata* (*Ptm*), which cause net-form net blotch (NFNB) and spot-form net blotch (SFNB) symptoms in barley, respectively.

To date, six bi-parental mapping studies have been conducted to discover genomic regions accounting for avirulence/virulence of *Ptt/Ptt* populations^24–29^ while other studies have detected QTLs responsible for the virulence in *Ptm/Ptm*^30^ and *Ptt/Ptm* hybrid mapping populations^31^. Two GWAS have also been conducted to identify genomic regions responsible for the virulence of *P. teres* in *Ptt*^29^ and *Ptm*^32^ populations. These mapping studies have identified QTLs responsible for the virulence of many globally grown barley cultivars like Beecher, Harbin, Kombar, Rika and Skiff across all 12 chromosomes in the *P. teres* genome, demonstrating the complexity of the *P. teres*-barley pathosystem. The current study aimed to 1. identify virulence QTL using a MP-NAM population developed by crossing *Ptt* isolates that are virulent and avirulent on barley cultivars Skiff and Prior, and 2. determine the QTL detection power of a MPP population i.e. compare MP-NAM versus bi-parental mapping.

## Materials and methods

### Biological materials

Four *Ptt* populations (HRS11093xHRS09127, HRS11093xHRS10136, NB81xHRS09127 and NB81xHRS10136) consisting of 403 progeny isolates in total were developed by crossing four *Ptt* isolates (Table 1). Crosses were made as indicated in Dahanayaka et al.^51^. Four barley cultivars, Beecher, Commander, Prior, and Skiff, obtained from the Department of Agriculture and Fisheries in Queensland, were used in the phenotypic assays for the QTL analyses.

**Table 1:** Details of the four bi-parental mapping populations.

Cross	Isolate Parent 1	Mating type	Disease reaction score		Isolate Parent 2	Mating type	Disease reaction score		Number of ascospores used for genetic maps
			Prior	Skiff			Prior	Skiff	
1	HRS11093	*MAT1-1*	10	1.5	HRS09127	*MAT1-2*	1	9.8	94
2	HRS11093	*MAT1-1*	10	1.5	HRS10136	*MAT1-2*	1	9	94
3	NB81	*MAT1-1*	10	1.5	HRS09127	*MAT1-2*	1	9.8	120
4	NB81	*MAT1-1*	10	1.5	HRS10136	*MAT1-2*	1	9	95

### Genotyping by DArTseq

Progeny and parental cultures were grown on half-strength potato dextrose agar (PDA) medium (20 g/L PDA; Biolab Merck, Darmstadt, Germany) at 22 °C for 10 days. The mycelia of the progeny and parental isolates were scraped and freeze dried for 48 h. Freeze dried samples were sent to Diversity Arrays Technology Pty. Ltd. (Canberra, ACT, Australia) for DNA extraction and DArTseq™.

### Phenotyping of the progeny isolates

Phenotyping of the progeny isolates was performed following a completely randomised design in a controlled environment room at the University of Southern Queensland, Australia, with three replicates, as described in Dahanayaka et al.^31^. The four barley cultivars (Beecher, Commander, Prior, and Skiff) were grown in pots with 5 cm diameter and 14 cm height with each pot containing four plants each of the four barley cultivars. Plants were grown at day (12 h) and night (12 h) temperature of 23 ± 1 °C and 17 ± 1 °C respectively, at 75% humidity for 14 days. Barley cultivars Beecher and Commander were used as the resistant and susceptible control, respectively.

The conidial suspensions for inoculations were prepared as described in Dahanayaka et al.^31^. Three millilitres of the suspension diluted to 10,000 conidia/mL were used per pot for inoculations 14 days after sowing. The parental isolate HRS10136 was used as the control isolate for each inoculation experiment to monitor disease reaction score differences across different experiments. After inoculation pots were incubated in the dark for 24 h at 95% humidity with a temperature of 20 ± 1 °C. Plants were then transferred to the controlled environment room with the same environmental conditions as mentioned above. Nine days after inoculation, disease reaction scores on the second leaf of the barley plants were recorded (scale 1–10, where 1 represents no symptoms 10 represents dead leaves)^33^.

### Genetic map construction

Individual genetic maps for four populations were constructed using SilicoDArT and SNP marker data obtained from DArTseq™. Both markers were filtered using 10% as the cut-off value for the minimum amount of missing data per isolates. Non-polymorphic markers were removed along with markers deviating from the 1:1 segregation ratio. Clonal isolate pairs of each progeny were identified using the *clonecorrect* function in poppr package version 2.8.3^34^ in R version 3.0.2^35^ and one clonal isolate from each pair was removed. SilicoDArT and SNP markers were grouped into linkage groups using the *make linkage groups* function in MapManager QTXb20 version 2.0^36^ with a *p* = 0.05 search linkage criterion. Markers were ordered using RECORD^37^ and the final genetic map of each population was obtained after manual map curation^38^. The marker order of the linkage groups was confirmed by aligning marker positions to the *Ptt* reference genome, W1-1 (BioSample SAMEA4560035, BioProject PRJEB18107) using the bowtie2^39^ function in Galaxy.

### QTL detection by bi-parental mapping populations

The composite interval mapping method in Windows QTL Cartographer version 2.5^40^ was used to identify QTLs associated with virulence in the bi-parental mapping populations. Experiment-wise LOD threshold values for each phenotypic trait were estimated at the 0.05 significance level based on 1,000 permutations^41,42^. Additive effects and the phenotypic variances explained by each QTL (R^2^) were calculated by Windows QTL Cartographer version 2.5. MapChart version 2.32^43^ was used to draw the QTL map.

### QTL detection by MP-NAM population

The *mppR* package^44^ available in RStudio was used to detect QTLs associated with virulence in *Ptt* using the MP-NAM population. The genotypic and phenotypic data for the four bi-parental populations were combined to represent a single MP-NAM population. The final data set was quality filtered with 5% as the threshold minimum allele frequency for markers and 25% as the maximum missing data per isolate. This resulted in 1135 SilicoDArT and SNP markers from DArTseq™ and 399 progeny isolates being retained for the MP-NAM QTL analysis. QTL detection was carried out using cross-specific, bi-allelic and parental QTL effect models available in the *mppR* package. Significant thresholds for each trait were calculated using 1000 permutations. Cofactors were selected using the simple interval mapping method and multi-QTL model searches were implemented by composite interval mapping to detect QTLs in different types of effect models; bi-allelic (two alleles at the QTL position with consistent effect through the QTL position), cross-specific (at the QTL the allelic effects can be different in every cross), and parental (every parent carries a different allele with a consistent effect in every cross). The regression coefficients (R^2^) for each QTL were detected. Cross validations for the detected QTLs were carried out to assess the QTL effect in a pseudo-independent population to confirm the putative QTLs.

### Comparison between bi-parental and MP-NAM populations for QTL detection

The power of QTL detection of bi-parental versus MP-NAM populations was compared by determining the number of QTLs identified with each set. Thus, results produced by the three MP-NAM QTL models were compared with results obtained from the four individual bi-parental mapping populations. Fifty isolates from each bi-parental population were randomly selected and pooled to create a smaller bi-parental and MP-NAM populations and the QTL analysis was conducted with Windows QTL Cartographer and the *mppR* package, respectively, following the same criteria as above. The analyses were repeated three times using three different sub-datasets (Run_1, Run_2 and Run_3). Each sub-dataset contained 50 randomly selected isolates from each bi-parental population.

### Genetic diversity of the population

Principal analysis of coordinates (PCoA) and a neighbor-net network were used to detect the overall diversity structure and the genetic distribution of the isolates of each population. PCoA for the progeny isolates used in the MP-NAM population was conducted in DARwin v.6.0^45^ using Euclidean distances with five principal coordinates. Neighbor-net network was built for the MP-NAM population in SplitsTree version 4.13^46^ with 1000 bootstrap. The Neighbor-net network was built based on the method described by Bryant and Moulton^47^ using neighbor joining algorithm^48^.

### Plant ethical approval

Experimental research and field studies on plants (either cultivated or wild), including the collection of plant material, must comply with relevant institutional, national, and international guidelines and legislation.

## Results

### DArTseq analysis

A total of 4809 SNPs and 9810 SilicoDArT were obtained from DArTseq™. After quality filtering of markers for 10% missing values, non-polymorphism and segregation distortion (1:1), 1241, 1102, 568 and 1168 SNPs and SlicoDArT markers were retained for the HRS11093xHRS09127, HRS11093xHRS10136, NB81xHRS09127 and NB81xHRS10136 populations, respectively and used for the phenotypic evaluation and genetic map construction (Table 1).

### Phenotyping of the progeny isolates

**Figure 1:**
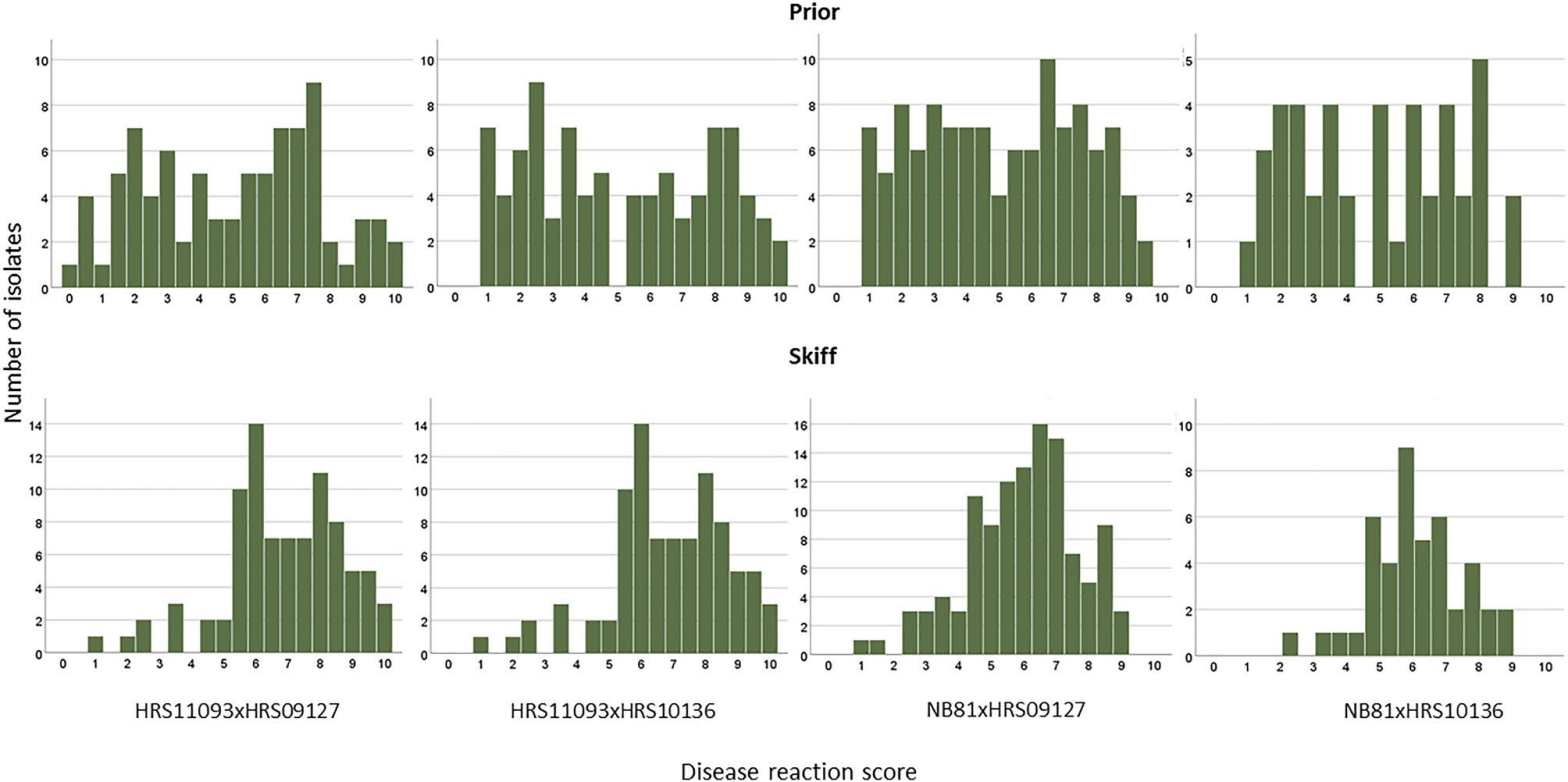
Disease reaction scores of progeny isolates from bi-parental mapping populations on barley cultivars Prior and Skiff used in QTL analyses.

Disease reaction scores of the progeny isolates of the populations on barley cultivars Prior and Skiff ranged from avirulent to virulent (Fig. 1 and Table 2). Some of the isolates used in the genetic map construction did not produce conidia for phenotyping, hence the difference in numbers. Disease reaction scores of these progeny isolates were skewed on the resistant control Beecher and the susceptible control Commander towards avirulent and virulent, respectively, as expected (data not shown); hence, phenotypic data obtained from Beecher and Commander were not used for QTL analyses.

**Table 2:** Disease reaction scores for barley cultivars Prior and Skiff used for QTL analyses.

Cross	Number of isolates^a^	Mean^b^	SE^c^	SD^d^	Skewness	Minimum^e^	Maximum^f^
Prior							
HRS11093xHRS09127	85	5.0	0.29	2.65	− 0.05	0.0	10.0
HRS11093xHRS10136	88	5.1	0.30	2.81	0.11	1.0	10.0
NB81xHRS09127	115	5.0	0.22	2.44	− 0.02	1.0	9.3
NB81xHRS10136	44	4.9	0.36	2.37	0.05	1.0	9.2
MP-NAM^g^	332	5.0	0.14	2.58	0.31	0.0	10.0
Skiff							
HRS11093HRS09127	85	6.2	0.22	2.03	− 0.97	0.0	10.0
HRS11093xHRS10136	88	6.8	0.19	1.84	− 0.66	1.0	10.0
NB81xHRS09127	115	5.9	0.16	1.69	− 0.39	1.0	9.2
NB81xHRS10136	44	6.1	0.22	1.43	− 0.26	2.3	9.0
MP-NAM	332	6.3	0.10	1.82	− 0.59	0.0	10.0

^a^Number of isolates with phenotypic data.

^b^Mean disease reaction score.

^c^Standard error.

^d^Standard deviation.

^e^Minimum disease reaction score.

^f^Maximum disease reaction score.

^g^Multi-parental Nested association mapping population.

### Genetic map and bi-parental QTL analyses

The genetic maps of the four bi-parental populations consisted of 12–16 linkage groups spanning from 2029 to 2683 cM (Table 3). The average distance between flanking markers for the four populations ranged from 4.56 to 6.29 cM. The physical distance to genetic map distance ratio for the four populations with respect to the *Ptt *reference genome W1-1 ranged from 19.29 to 25.51 kb/cM.

**Table 3:** Genetic map information for bi-parental mapping populations.

Cross	Number of non-redundant markers	Number of linkage groups	Size of the genetic map cM	Average distance between flanking markers	Physical distance to genetic map distance ratio (kb/cM)
HRS11093xHRS09127	428	16	2415	5.64	21.43
HRS11093xHRS10136	496	13	2683	5.40	19.29
NB81xHRS09127	445	12	2029	4.56	25.51
NB81xHRS10136	390	16	2456	6.29	21.07

**Figure 2:**
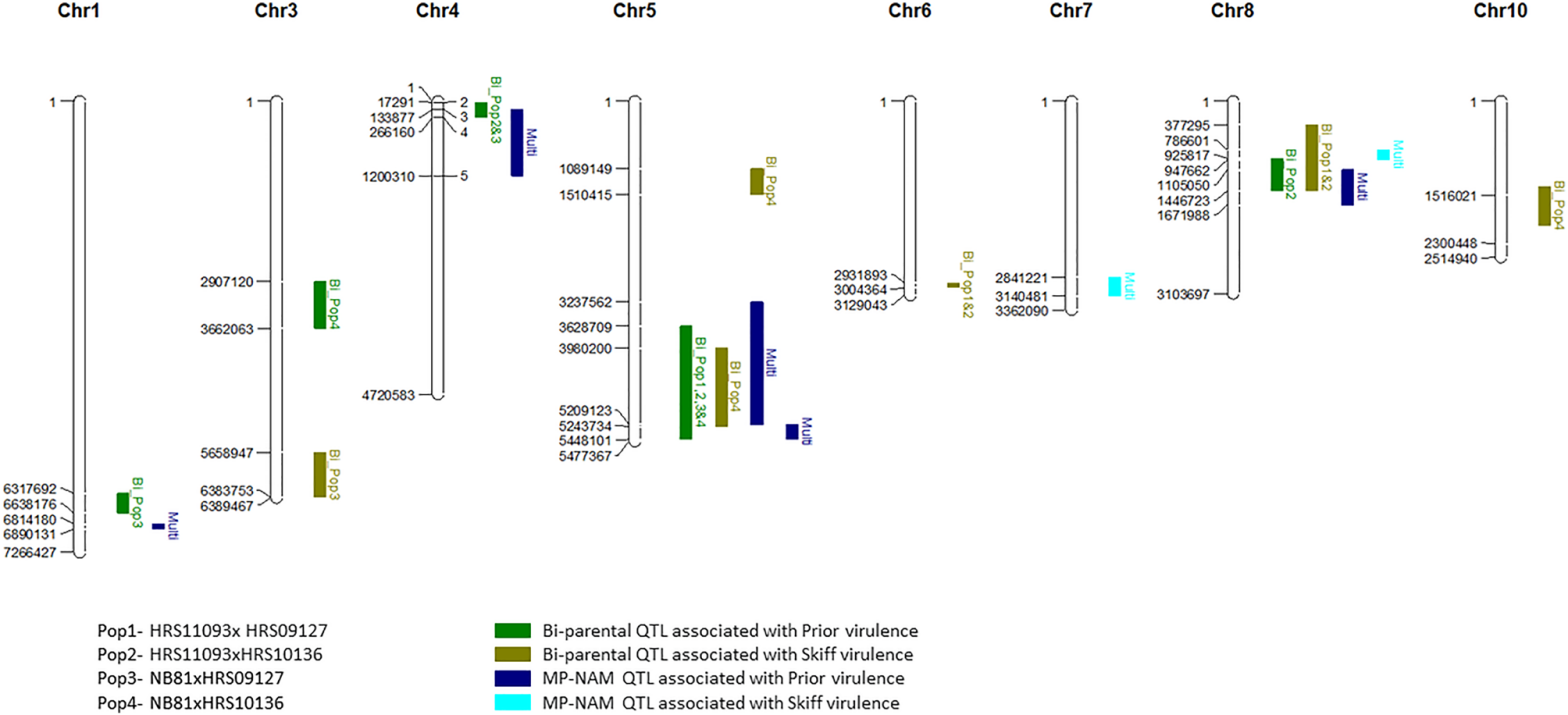
QTLs identified from bi-parental (Bi) and multi-parental (Multi) mapping populations for barley cultivars Prior and Skiff aligned to reference genome W1-1. QTLs are displayed to the right of the chromosome and map distances in base pairs on the left.

Results of the bi-parental mapping population QTL analysis are presented in Table 4 and Fig. 2. Between one to three QTLs associated with Prior or Skiff virulence were detected with individual bi-parental populations. A highly significant QTL associated with the Prior virulence was identified on chromosome 5 in all four populations. Significant LOD values for the QTLs ranged from 6.9 to 20.0 across the different populations. The phenotypic variance explained by the QTLs ranged from 33 to 63%. The most significant QTL identified for the Skiff virulence was located on chromosome 3 with a LOD score of 11.0 and 27% of the phenotypic variance explained. This QTL was only identified in the NB81xHRS09127 population. A QTL associated with Skiff virulence was detected on chromosome 5 in the NB81xHRS10136 population with a LOD score of 6.4 and 33% of the phenotypic variation explained. This QTL was located in the same region as the Prior virulence QTL.

**Table 4:** List of QTLs for virulence to barley cultivars Prior and Skiff identified from bi-parental mapping populations.

QTLs detected from the original bi-parental mapping populations (*N* = ~ 100)									QTLs detected from the subsets of bi-parental mapping populations (*N* = 50)					
Cross ID	Chr^a^	Marker names^b^		*Ptt* reference genome position W1-1 (bp)		LOD^c^	R^2d^	Parent^e^	Run_1		Run_2		Run_3	
		Start marker	End marker	Start	End				LOD^c^	R^2d^	LOD^c^	R^2d^	LOD^c^	R^2d^
Prior														
HRS11093xHRS09127	5	28949553	36348141	3628709	5448101	18.0	63	HRS11093	8.0	39	7.8	37	8.9	32
HRS11093xHRS10136	4	41806163	28945166	17291	133877	4.2	10	HRS11093	NA	NA	NA	NA	NA	NA
	5	36351297	28949678	5117,957	5187816	12.0	38	HRS11093	12.0	69	11.8	70	NA	NA
	8	28946536	74667498	925817	1446723	3.6	9	HRS11093	NA	NA	NA	NA	NA	NA
NB81xHRS09127	1	28945819	28949638	6317692	6638176	3.6	6	NB81	NA	NA	NA	NA	NA	NA
	4	36353507	28,949623	17291	266160	5.4	8	NB81	6.0	17	NA	NA	NA	NA
	5	28947023	28946415	3980200	5195868	20.0	39	NB81	14.0	50	NA	NA	5.6	50
NB81xHRS10136	3	28948412	28948179	2907120	3662063	4.3	19	NB81	4.0	17	NA	NA	11.0	28
	5	28947023	36348141	3980200	5243734	6.9	33	NB81	8.0	39	NA	NA	9.8	22
Skiff														
HRS11093xHRS09127	6	70125623	70125623	NA	NA	3.4	14	HRS01927	NA	NA	NA	NA	NA	NA
	8	36352197	28949798	377295	1369307	3.4	12	HRS01927	NA	NA	NA	NA	3.4	19
HRS11093xHRS10136	6	28945564	36347277	2931893	3004364	5.0	25	HRS10136	NA	NA	NA	NA	3.2	16
	8	28946536	74667498	925817	1446723	4.6	18	HRS10136	NA	NA	NA	NA	NA	NA
NB81xHRS09127	3	41806520	28949264	5658947	6383753	11.0	27	HRS01927	8.0	49	NA	NA	7.8	49
	10	36347247	28949840	1373217	2000757	3.4	8	HRS01927	NA	NA	NA	NA	NA	NA
NB81xHRS10136	5.1	36348141	28947023	3980200	5243734	6.4	33	HRS10136	6.0	29	NA	NA	NA	NA
	5.2	28945347	28946676	1089149	1510415	5.5	20	HRS10136	5.5	15	4.6	37	NA	NA

NA, not available.

^a^Chromosome number according to W1-1 reference genome.

^b^Flanking marker names of the QTL.

^c^Logarithm of the odds.

^d^Phenotypic variation described by the respective QTL.

^e^Parental isolate contributing the QTL.

### MP-NAM QTL analysis

QTL analysis of the MP-NAM population was carried out based on the bi-allelic, cross-specific and parental QTL effect models using 399 progeny isolates. Maximum of five and three QTLs were identified for the Prior and Skiff virulence, respectively. All models identified a QTL on chromosome 5 for Prior virulence with LOD scores ranging from 30.9 to 36.7 (Table 5). The phenotypic variance explained by the QTLs ranged from 14 to 39%. With both the cross-specific and parental models the QTL on chromosome 5 was split into two different QTLs. For Skiff virulence, three QTLs were detected across three different chromosomes with LOD scores ranging from 3.8 to 9.9 and phenotypic variance explained by these QTLs ranged from 4 to 13% across the different models. The same QTL was detected on chromosome 3 across all three models.

**Table 5:** List of QTLs for virulence to barley cultivars Prior and Skiff identified from multi-parental mapping populations.

QTLs detected from the original MP-NAM mapping populations (*N* = 399)								QTLs detected from the subsets of MP-NAM mapping populations (*N* = 200)								
QTL model	Chr^a^	Peak marker name^b^	QTL position in reference genome W1_1 (bp)		LOD^c^	R^2d^	Range cM^e^	Run_1			Run_2			Run_3		
			Start	End				LOD^c^	R^2d^	Range cM^e^	LOD^c^	R2^d^	Range cM^e^	LOD^c^	R2^d^	Range cM^e^
Prior																
Biallelic	1	28946148	6814180	6890131	6.1	4	26.0	3.6	5	26.4	4.3	3	9.4	NA	NA	NA
Biallelic	4	28948011	17291	1200310	4.9	6	17.0	3.3	5	17.3	3.9	6	17.3	4.5	13	17.3
Biallelic	5	28949358	5209123	5448101	36.7	39	0.3	22.6	39	0.7	20.5	40	0.7	26	54	0.7
Cross-specific	1	28946148	6596840	6890131	3.2	2	36.5	4.0	6	1.4	4.1	4	1.4	NA	NA	NA
Cross-specific	4	36345919	133877	1011565	7.7	8	6.1	4.2	6	17.3	6	10	6.7	5.3	7	17.3
Cross-specific	5	28949553	3237562	5209123	3.6	2	42.6	NA	NA	NA	3.3	3	42.6	NA	NA	NA
Cross-specific	5	36347644	5209123	5448101	30.9	16	0.3	20.8	40	0.34	17.2	19	0.3	24.4	48	0.3
Cross-specific	8	28948394	1105050	1671988	3.5	3	18.9	NA	NA	NA	NA	NA	NA	NA	NA	NA
Parental	1	28946148	6814180	6890131	3.4	3	26.4	NA	NA	NA	4.3	4	1.4	NA	NA	NA
Parental	4	36345919	133877	1011565	7.2	6	6.1	3.5	5	17.3	5.8	10	6.7	5.2	7	17.3
Parental	5	28949553	3237562	5209123	4.2	3	42.6	NA	NA	NA	3.2	2	42.6	NA	NA	NA
Parental	5	36347644	5209123	5448101	31.0	14	0.34	20.4	38	0.34	17.9	19	0.3	24	45	0.3
Skiff																
Biallelic	3	36347042	N/A	N/A	8.9	12	1.8	5.0	10	0.36	4.9	12	2.5	4.3	10	1.8
Cross-specific	3	28949870	N/A	N/A	9.9	13	2.5	4.6	11	2.4	3	11	3.6	3.5	11	3.6
Cross-specific	7	28947398	2841221	3140481	5.2	7	18.3	3.4	8	18.2	NA	NA	NA	NA	NA	NA
Cross-specific	8	28946174	786601	947662	3.8	4	6.7	NA	NA	NA	NA	NA	NA	NA	NA	NA
Parental	3	28947129	N/A	N/A	9.6	12	2.7	4.5	15	2.4	3.4	12	13.3	3.5	10	3.9
Parental	7	28947398	2971153	3140481	4.1	5	8.2	NA	NA	NA	NA	NA	NA	NA	NA	NA
Parental	8	28946174	786601	947662	3.8	4	6.7	3	7	12	NA	NA	NA	NA	NA	NA

NA, not available.

^a^Chromosome number according to W1-1 reference genome.

^b^Peak marker name of the QTL.

^c^Logarithm of the odds.

^d^Phenotypic variation described by the respective QTL.

^e^Range of the QTL.

### Comparison of bi-parental mapping to MP-NAM mapping

The maximum number of QTLs detected using the MP-NAM population (*N* = 399) was eight with the cross-specific QTL model, compared to a maximum of five detected when using an individual bi-parental mapping population (*N* =  ~ 100). The strength of MP-NAM versus bi-parental mapping QTL analyses was further tested using populations containing fewer individuals, i.e. *N* = 200 for MP-NAM and *N* = 50 for each of the bi-parental populations. Fifty individuals were randomly chosen for each of the population and for three different runs. Out of the three runs (Run_1, Run_2 and Run_3), Run_1 obtained the highest number of QTLs in the MP-NAM QTL analysis (Table 5). The QTLs detected with all three models for Prior virulence on chromosomes 4 and 5 in the original MP-NAM mapping population were also reported for all runs and models in the smaller MP-NAM population. Similarly, the chromosome 3 QTL associated with Skiff virulence was detected with all models and runs. Minor QTLs were only detected in some runs or not at all with some of the models, e.g. QTL on chromosome 8 associated with Prior virulence (Table 5). Using the bi-parental mapping populations only the QTL on chromosome 5 of the HRS11093xHRS09127 population was detected in all runs (Table 5). None of the QTLs associated with Skiff virulence were detected in all runs.

### Genetic diversity of the population

**Figure 3:**
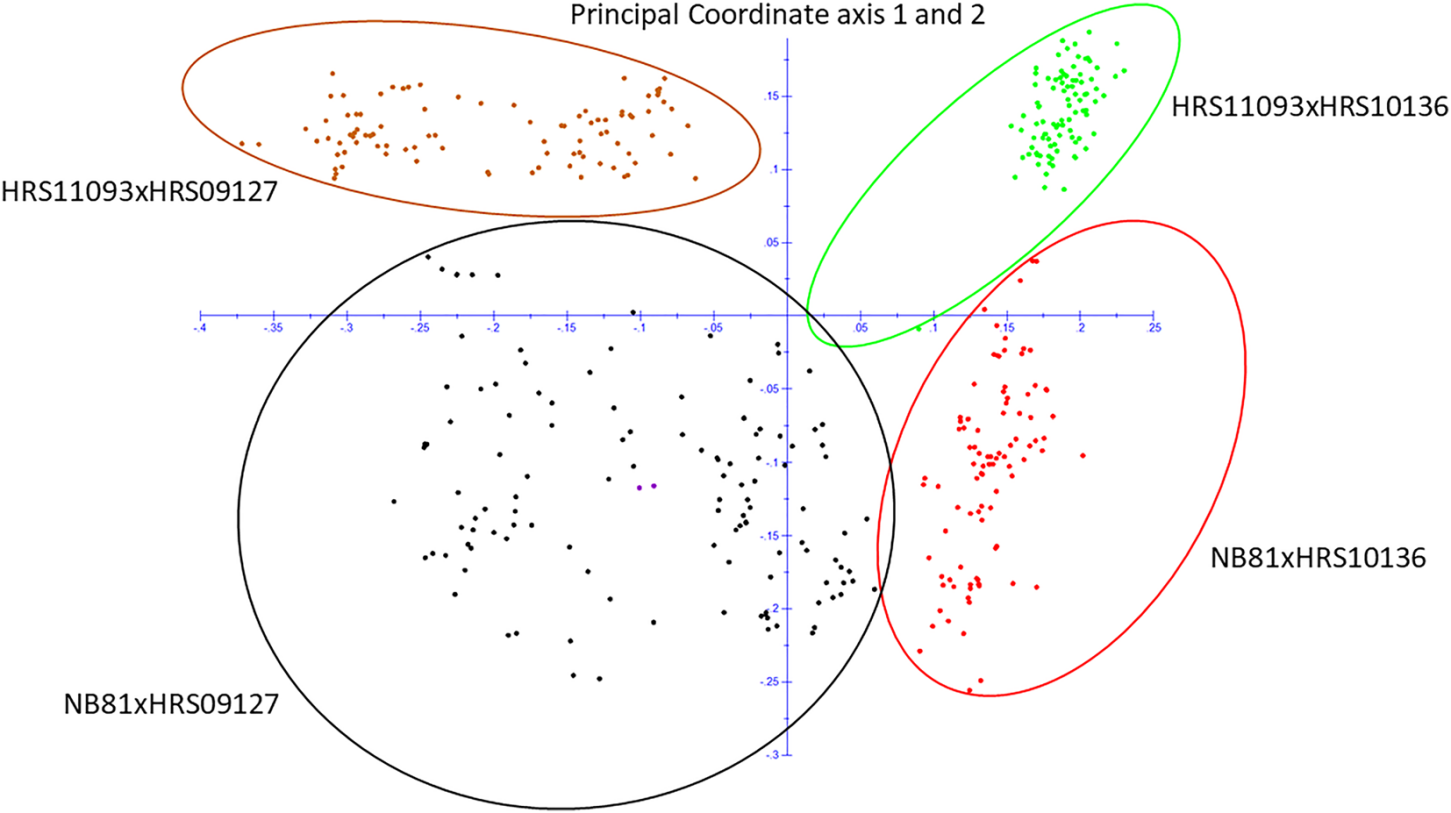
Principal analysis of coordinates for the MP-NAM population. Principal coordinate axis 1 and principal coordinate axis 2 explained 12 and 6%, respectively, for the genetic clusters.

**Figure 4:**
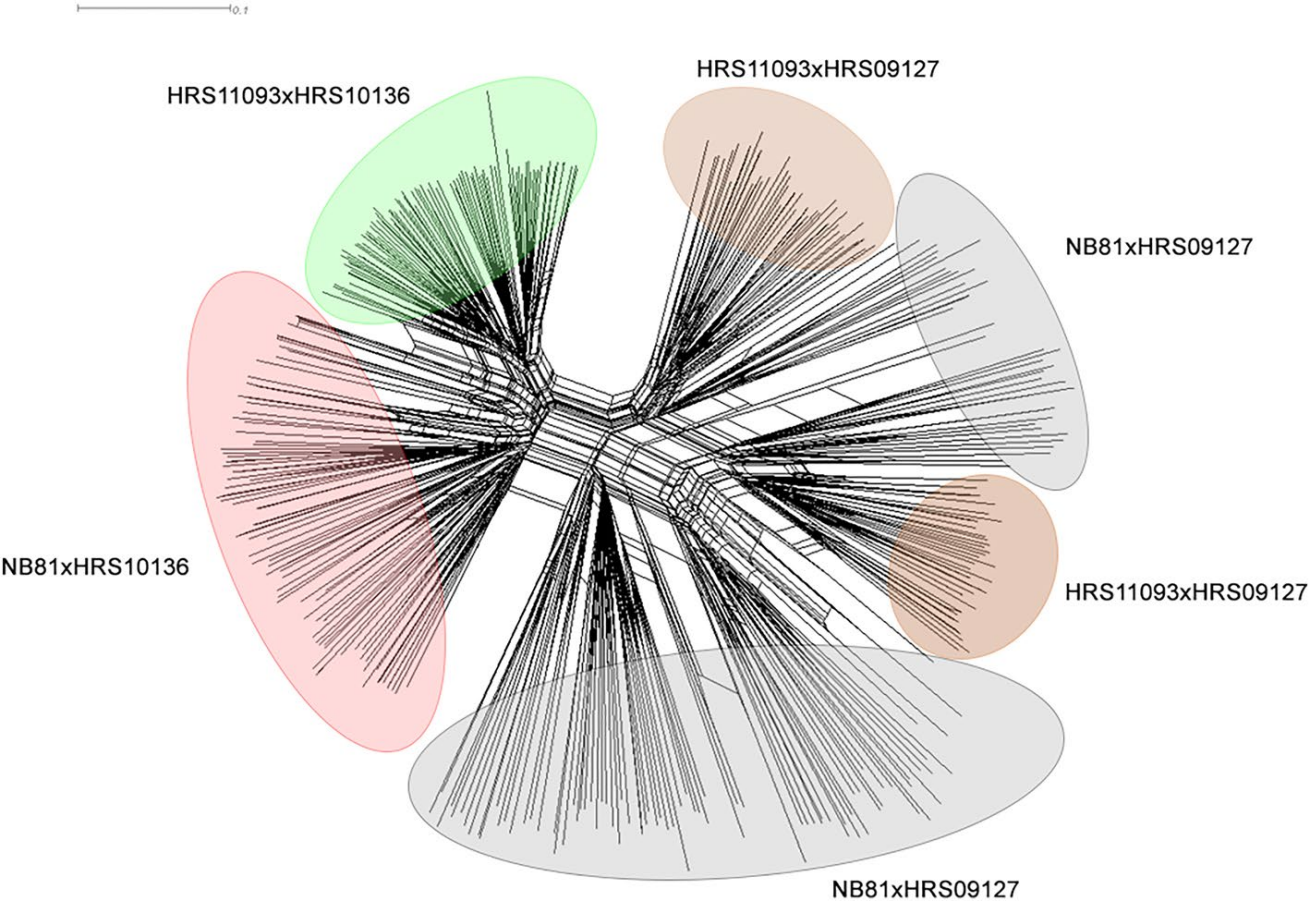
Neighbor-net network based on DArTseq marker for the MP-NAM mapping population.

The PCoA of 399 progeny isolates showed clear clustering of the four bi-parental populations as expected. The first and second principal coordinate axes (PCoA1 and PCoA2) explained 12 and 6% of the variance, respectively, and separated clusters by population. Out of the four populations, NB81xHRS09127 showed the highest genetic diversity within the population while HRS11093xHRS10136 showed the lowest (Fig. 3). The neighbor-net network consisted of two main groups. One group contained progeny isolates from HRS11093xHRS10136 and NB81xHRS10136 while the other group contained progeny isolates from HRS11093xHRS09127 and NB81xHRS09127. The neighbor-net network constructed for the MP-NAM population was highly reticulated as expected (Fig. 4).

## Discussion

Multi-parental mapping populations have been used for many crops to detect QTLs as they have been useful in providing insight into the genetic architecture of complex phenotypes like grain yield, plant height and biotic and abiotic resistance amongst other phenotypes^44^. However, as per the authors’ knowledge usage of MPPs to identify QTLs in fungi has not previously been reported. This the first study to use a MP-NAM population to identify QTLs in a fungus.

Bi-parental mapping populations have been extensively used in the detection of QTLs in crops^14,19^. However, there are limitations with bi-parental mapping populations compared to MPPs in the successful detection of significant QTLs^11,16^. The principal limitations of bi-parental mapping populations are low genetic diversity resulting from genetic bottlenecking due to the choice of two parents and limited events of effective recombination to develop a precise genetic map. These limitations reduce the number of QTLs captured by bi-parental mapping populations.

The majority of NAM populations have been developed by crossing one reference parent with several other donor parents to obtain a diverse genetic background^49^. The NAM population used in this study was developed using more than one reference parent. The genetic principle behind the MP-NAM analysis used in this study is based on bi-parental populations and diversity panels. Bi-parental population analysis has the confounding effect of population structure, which is avoided when using MP-NAM analysis. Another advantage of MP-NAM populations is the detection of multiple alleles in the same QTL along with rarer and more diverse alleles.

Two out of the four parental isolates used in our mapping populations demonstrated high and low virulence on barley cultivars Prior and Skiff, respectively, while the other two demonstrated low and high virulence on Prior and Skiff, respectively. An increased number of recombination events occurring in a fungal population increases the genetic diversity of the population^50^. Using more than one parental isolate for the desired phenotype increases genetic diversity of the population as shown in the neighbour-net network and PCoA analysis in the current study and captures QTLs associated with the desired phenotype from more than one parent. As shown in this study, one to three QTLs were detected that were associated with Prior virulence using bi-parental mapping populations while five QTLs were detected using the MP-NAM population. For Skiff virulence, only two QTLs were reported for each bi-parental mapping population while three QTLs were detected for the MP-NAM population, thus confirming that the MP-NAM QTL detection method can detect a greater number of QTLs than the bi-parental method.

Most QTLs identified in the four bi-parental mapping populations of this study were co-localised, e.g. QTLs on chromosomes 4 and 5 identified for the Prior virulence. In populations HRS11093xHRS09127 and HRS11093xHRS10136 the contributing parent for the QTL on chromosome 5 was HSR11093 and in NB81xHRS09127 and NB81xHRS10136, the contributing parent for the QTL was NB81 suggesting that these isolates possess the same genomic regions on chromosome 5 associated with Prior virulence. QTLs associated with Prior virulence identified on chromosome 1, 3, and 8 of population NB81xHRS09127, NB81xHRS10136 and HRS11093xHRS10136, respectively, were unique QTL, not detected in the other populations. The QTL on chromosome 8 associated with Skiff virulence of bi-parental mapping population was also co-located with the QTL on chromosome 8 for Prior virulence. The QTL associated with Skiff virulence on chromosome 5 also co-located with the QTL associated with Prior virulence on chromosome 5. Co-localization of QTL associated with Prior and Skiff virulence could be due to either the presence of tightly linked genes in the same genomic region or lack of effective events of recombination between the two regions to identify the two genes as separate QTLs.

The QTLs identified on chromosomes 1, 4 and 5 for Prior virulence from the multi-parent population based on biallelic, cross-specific and parental models were located at the same genomic regions. The QTL detected on chromosome 5 for Prior virulence based on the biallelic model was a broad QTL. This QTL was detected as two QTLs in the cross-specific and parental models suggesting that there are two QTLs in this genomic region. This also suggests that the cross-specific and parental models may have better resolution power than the biallelic model. All QTLs associated with Prior virulence and QTL on chromosome 8 for Skiff virulence of the MP-NAM population were co-located with the QTLs identified by the four bi-parental mapping populations. Co-localisation of these QTLs validates the robustness of the QTLs identified in our study.

QTLs identified in our study were also co-localised with QTLs reported in previous bi-parental and GWAS studies. The location of the QTL identified on chromosome 5 for Prior virulence was similar to the QTLs *AvrHar*^24^*, PttBee2* (Koladia et al.^28^)*, PttBee_5, PttSki_5, QTL11*, *QTL12* (Martin et al.^4^) and *USQV5* (Dahanayaka et al.^31^) identified previously through bi-parental and GWAS studies. The QTL, *PttBee2*^28^ and *PttBee_5*^4,29^ associated with Beecher virulence were co-localised with our QTL on chromosome 5 associated with Prior virulence. The four parental isolates used in the current study were avirulent on barley cultivar Beecher and hence, used as the resistant control. Co-localisation of the Prior virulence QTL and the Beecher virulence QTL suggests that this genomic region codes for a gene/genes which would induce multiple reactions in different *Ptt* isolates. This may be due to the presence of different alleles of the same gene in different *Ptt* isolates or to possessing more than one virulence gene in the same genomic region. The QTL associated with Prior virulence on chromosomes 8 in the current study shared the same genomic region as *PttTif2*^28^. Three QTLs responsible for Skiff virulence detected in the current study on chromosomes 3, 7 and 10 were co-located with QTL *QTL7, PttPri_7*^4,29^ and *VR2*^27^, respectively.

Comparison of the bi-parental and MP-NAM analyses revealed that MP-NAM QTL analysis had greater power to detect QTLs than bi-parental QTL mapping, even with a low number of progeny isolates. The number of progeny obtained from crossing fungal isolates can vary^51^. In some instances, crosses may produce insufficient numbers of ascospores for bi-parental mapping analyses. On such occasions, a MPP like MP-NAM is a useful alternative method for identifying QTLs in populations with low progeny numbers, where inter-connected multiple populations are available.

In conclusion, this study reported the first MPP QTL analysis conducted with a fungal population. The bi-parental mapping QTL analyses detected one to three QTLs associated with Prior virulence and two with Skiff virulence for the four populations developed from crossing *P. teres* f. *teres* isolates. The MP-NAM population, developed by combining the bi-parental mapping populations, detected five and three QTLs for Prior and Skiff virulence, respectively. The comparison of bi-parental and MP-NAM analyses revealed that QTL detection power of a MP-NAM population was greater than that of a bi-parental fungal mapping population even with a low number of progeny.

## Data Availability

The datasets generated during and/or analysed during the current study are available from the corresponding author on request.
